# Transient expression of Wnt5a elicits ocular features of pseudoexfoliation syndrome in mice

**DOI:** 10.1371/journal.pone.0212569

**Published:** 2019-03-06

**Authors:** Yong Yuan, Ursula Schlötzer-Schrehardt, Robert Ritch, Mindy Call, Fred B. Chu, Fei Dong, Taylor Rice, Jianhua Zhang, Winston W.-Y. Kao

**Affiliations:** 1 Crawley Vision Research Laboratory, Department of Ophthalmology, College of Medicine, University of Cincinnati, Cincinnati, Ohio, United States of America; 2 Department of Ophthalmology, Universitätsklinikum Erlangen, Friedrich-Alexander-Universität Erlangen-Nürnberg, Erlangen, Germany; 3 Einhorn Clinical Research Center, New York Eye and Ear Infirmary of Mount Sinai, New York, NY, United States of America; 4 Cincinnati Eye Institute, Cincinnati, Ohio, United States of America; Bascom Palmer Eye Institute, UNITED STATES

## Abstract

**Purpose:**

Pseudoexfoliation (PEX) syndrome is an age-related systemic disease with ocular manifestations. The development of animal models is critical in order to elucidate the cause of the disease and to test potential treatment regimens. The purpose of this study is to report phenotypes found in mouse eyes injected with Adenovirus coding Wnt5a. Some of the phenotypes resemble those found in PEX patients while others are different.

**Methods:**

Recombinant Adenovirus coding Wnt5a or green fluorescent protein (GFP) were injected into mouse eyes. Two months after the injection, eyes were examined for PEX phenotypes using slit lamp, fluorescence stereomicroscope, histological staining, immunostaining and transmission electron microscope.

**Result:**

Certain ocular features of PEX syndrome were found in mouse eyes injected with recombinant Adenovirus coding Wnt5a. These features include accumulation of exfoliation-like extracellular material on surfaces of anterior segment structures and its dispersion in the anterior chamber, saw-tooth appearance and disrupted basement membrane of the posterior iris pigment epithelium, iris stromal atrophy and disorganized ciliary zonules. Ultrastructure analysis of the exfoliation material revealed that the microfibril structure found in this model was different from those of PEX patients.

**Conclusion:**

These features, resembling signs of ocular PEX syndrome in patients, suggest that new information obtained from this study will be helpful for developing better mouse models for PEX syndrome.

## Introduction

Pseudoexfoliation (PEX) syndrome is an age-related systemic disease characterized by the accumulation of an extracellular fibrillar material in the eyes, skin, lungs, heart, kidneys and other organs [1,2]. PEX syndrome is diagnosed clinically by slit lamp examination showing ‘dandruff-like’ material deposits in the anterior chamber, and on the anterior lens capsule and the pupillary border of the iris. About 30% of patients with PEX syndrome will progress to glaucoma within 7 years [3]. It is believed that obstruction of the outflow pathway by a combination of exfoliation material (XFM) and pigment leads to secondary open-angle glaucoma [4], though the pathogenesis of the disease itself is still uncertain [5,6]. Further associated clinical signs and potential complications include angle-closure glaucoma, cataract, phacodonesis and lens subluxation due to weakened ciliary zonules, insufficient mydriasis, saw-tooth structure of the iris pigment epithelium, peripupillary transillumination defects due to dispersion of pigment, iris stromal atrophy, iris vasculopathy associated with blood-aqueous barrier defects, formation of posterior synechiae as well as corneal endothelial decompensation[7]. PEX patients are also prone to develop ocular surface diseases, such as dry eye. PEX syndrome causes decreased goblet cell density in the conjunctiva and reduction of tear film functions [8,9].

Over the past 20 years, several theories have emerged to explain the etiology of the disease. First, PEX syndrome has been described as a fibrillinopathy[7] since PEX fibrils predominantly contain components of elastic microfibrils, such as fibrillin-1[10], and latent transforming growth factor binding proteins (LTBP-1 and-2)[11]. Therefore, it has been suggested that the accumulation of exfoliation material results from excessive production and abnormal aggregation of elastic microfibrillar components. Abnormal production and deposition of microfibrils close to cell surfaces may affect basement membrane integrity, which can result in loss of barrier function. Impaired blood-aqueous barrier function has been reported in PEX syndrome eyes[12], resulting in a greater aqueous protein concentration and possibly the incorporation of serum proteins, such as amyloid P protein[13], apolipoprotein E (ApoE), complement factor C3 and clusterin[14], into the exfoliation material. Second, PEX syndrome shares common features with amyloid disorders [15]. Amyloid P protein and ApoE are found in both exfoliation material and amyloid-beta (Aβ) aggregates and Congo red-positive material has been found in the aqueous humor of PEX syndrome patients [16–18]. Third, aberrations in lysosomal/autophagic functions have been reported in cultured cells derived from PEX syndrome patients [19]. It is has been suggested that misfolded proteins escaping the intracellular degradation pathway may give rise to stable extracellular fiber aggregates.

Genetic analysis in the Icelandic population revealed that two single nucleotide polymorphisms of the lysyl oxidase-like 1 gene (*LOXL1*) were found in 99% of affected patients. This was the first gene found to be associated with PEX syndrome [20]. Follow-up studies confirming an association of *LOXL1* with PEX syndrome in multiple populations have also observed a high frequency of risk alleles in the general population[21], suggesting that *LOXL1* is necessary, but not sufficient to cause PEX syndrome. These observations suggest that transgenic mice harboring human risk alleles of *LOXL1* alone may not lead to disease manifestation and that screening for other genetic and environmental modifiers is required.

Currently, only one mouse line exhibits certain features of human PEX syndrome. The mice harbor a spontaneous mutation in the lysosomal trafficking regulator (*LYST*) gene [22]. LYST mice have iris transillumination defects and pigment dispersion in the anterior chamber. Fibrillin-positive exfoliation-like material has been identified in the anterior chamber. Interestingly, LYST mice do not develop glaucoma-related damage.

We have previously reported that the activation of noncanonical Wnt signaling is associated with a glaucomatous phenotype *in vivo* [23] and *in vitro* [24]. There are several Wnt signaling pathways, including canonical Wnt signaling and noncanonical Wnt signaling. A delicate balance between the two is key for achieving normal tissue structure and function. Canonical Wnt signaling is activated through the binding of Wnt ligands (such as Wnt2 and Wnt3a) to their co-receptors (LRP5 and LRP6). This releases β-catenin from a destruction complex and subsequently allows it to drive the expression of target genes by binding to TCF/LEF transcription factors [25]. On the other hand, noncanonical Wnt signaling is activated through the binding of Wnt ligands (mainly Wnt5a and Wnt4) to their receptors (ROR2 or VANGL2) [26]. This triggers the activation of a panel of small GTPases, such as RhoA, Rac1 and CDC42 [27]. Subsequently, Rho-associated kinase (ROCK) is activated and transduces the signal to myosin light chain to modulate cytoskeletal structure. ROCK and trabecular meshwork contractility have already been established as important regulators of intraocular pressure (IOP) [28]. Repressed canonical Wnt signaling has also been reported to be associated with IOP regulation [29], but it is still unknown if there is a link between repressed canonical Wnt signaling and the activation of ROCK in the trabecular meshwork [30]. When we studied the role of TGF-alpha on corneal epithelial homeostasis, we found secondary angle-closure glaucoma in this mouse line [23]. This unexpected observation led to the discovery that noncanonical Wnt signaling was activated in the trabecular meshwork. To test if Wnt5a alone can induce glaucoma, we injected Adenovirus-Wnt5a into the anterior chamber of the mouse eyes. While characterizing the retinal pathology of the model, we also noticed fibrillar debris in the anterior chamber of these eyes, prompting us to find out if this mouse model also has ocular features of PEX syndrome.

Using light and electron microscopy as well as immunohistochemistry, we observed several hallmarks of PEX syndrome in the eyes of these mice, including accumulation of exfoliation-like extracellular material on surfaces of anterior segment structures and its dispersion in the anterior chamber, saw-tooth appearance and disrupted basement membrane of the posterior iris pigment epithelium, iris stromal atrophy and disorganized ciliary zonules. Ultrastructure analysis of the exfoliation material revealed that the microfibril structure found in this model was different from those of PEX patients. These findings will be helpful for developing better mouse models for PEX syndrome.

## Methods

### Expression of Wnt5a in the anterior segment

Full-length mouse Wnt5a cDNA (pLNC Wnt-5aHA) was a gift from Jan Kitajewski (Addgene plasmid # 18032) [31]. This cDNA construct was used to generate the recombinant adenovirus vector according to a published protocol [32]. Briefly, Wnt5a cDNA was released from pLNC-Wnt5a and cloned into pTrackCMV, resulting in pTrackCMVWnt5a. This shuttle vector was linearized with the restriction enzyme PmeI and transformed into electrocompetent AdEasier cells. Recombinant adenovirus plasmids were selected by PacI restriction endonuclease digestion. Recombinant viruses were generated by transfecting HEK293 cells with the PacI-linearized recombinant plasmid. The virus was based on human adenovirus serotype 5 and had two separate CMV promoters to drive the expression of mouse Wnt5a and green fluorescent protein (GFP). Another recombinant adenovirus coding GFP alone (AdGFP) was used as control. Both AdGFP and AdWnt5a viruses were amplified and purified according to the protocol. For adenovirus injection, 4-month-old C57BL mice were anesthetized by intraperitoneal injection of ketamine hydrochloride (0.1 mg/gm body weight) and xylazine (0.01 mg/g body weight). A tunnel was made using a 30-gauge needle through the cornea of the left eye. The cornea was gently pressed to remove a small amount of aqueous humor from the eye, then, 2ul (about 10^9^pfu, plaque-forming units) of purified adenovirus was slowly injected into the anterior chamber using a Hamilton syringe. Three days after injection, eyes were evaluated with a fluorescent stereoscope and Optical Coherence Tomographic Imaging (Micron IV imaged guided OCT, Phoenix Research Labs). Eyes with apparent corneal opacity, blood in the anterior chamber, closed iridocorneal angle or GFP negativity were excluded from further analysis. Most eyes were collected two months (n = 6 each for AdGFP and AdWnt5a group) after injection for histopathologic and immunohistochemical analyses.

Animal care and use conformed to the ARVO Statement for the Use of Animals in Ophthalmic and Vision Research. All animal protocols were approved by the Institutional Animal Care and Use Committee (IACUC) of the University of Cincinnati. The mice used in this project were housed in AAALAC approved animal facilities within the University of Cincinnati/College of Medicine. Programs of animal husbandry, preventive medicine, and pre- and post-surgical care have been developed to assure that adequate veterinary care is provided at all times. Complete veterinary, diagnostic, and clinical support services were available.

### Slit-lamp examination

Slit lamp examinations were performed on a modified Topcon slit lamp. A beam splitter (BS7030-TOPCON) was installed in front of the eyepieces. An AccuBeam Video Adapter was attached to the beam splitter. A high definition digital camera was mounted on the Video Adapter. Animals were anesthetized and pupils were dilated, a vertical broad slit-lamp beam was placed on the pupillary margin. Still images were taken under the same beam intensity and exposure time.

### Stereomicroscopy and iris angiography

Fluorescein Ak-Fluor (10%; Akorn Pharmaceuticals), diluted with sterile 1 × DPBS (final concentration 10 mg/mL); was administered by bolus injection (50 μL) into the peritoneum of anesthetized mice. For iris angiography, the non-dilated eyes were observed under Axio Zoom.V16 stereo fluorescence microscope from Zeiss (Oberkochen, Germany).

### Histology and immunohistochemistry

Enucleated eyes were fixed in Davidson`s fixative overnight and dehydrated through graded ethanol steps, and embedded in paraffin according to standard protocols. Five micrometer thick sections were deparaffinized, rehydrated to PBS, and stained with Hematoxylin and Eosin (H&E) or Periodic acid–Schiff (PAS) reagents. To visualize the tissue structure of the iris, a de-pigmentation step was included, prior to staining, by treating the rehydrated slides with 10% hydrogen peroxide in PBS overnight. For immunofluorescence staining, an antigen retrieval step was performed by boiling the slides in citrate buffer for 10 minutes. The following primary antibodies were used: rabbit anti-collagen IV (Invitrogen, PA185320), rabbit anti-LOXL1 (Novus Biologicals, NBP182827), mouse anti-fibrillin (Millipore, MAB2641). Antibody detection was performed with Cy5 labeled goat anti-rabbit IgG (Invitrogen, A10523) and goat anti-mouse IgG (Invitrogen, A10524). Fluorescent images were taken using a Leica confocal microscope under 63x oil objective lens.

### Transmission electron microscopy

Electron microscopic examination was performed according to previous publications [33]. Briefly, enucleated mouse eyes were fixed in 2.5% glutaraldehyde in 0.15 mol/L phosphate buffer (pH 7.2) for 30 minutes. The eyes were then dissected at the equator and the lens removed to enable the fixative to enter into the eye. The two halves and the lens were stored in fixative at 4°C. After post-fixation in 2.0% buffered osmium tetroxide for 1 hour, the specimens were gradually dehydrated and embedded in epoxy resin (Epon). Ultrathin sections were cut, stained with uranyl acetate-lead citrate, and examined with a transmission electron microscope (LEO EM 906E; Carl Zeiss Microscopy GmbH, Oberkochen, Germany).

## Results

### Adenovirus-mediated transient expression of Wnt5a in the anterior segment of the mouse eye

To express Wnt5a in the anterior segment, Adenovirus-Wnt5a (Adwnt5a) was injected into the anterior chamber of adult mice. The AdWnt5a also co-expressed the GFP. Adenovirus-mediated gene expression was monitored by fluorescence stereomicroscope in live animals. As shown in S1 Fig, three days after the injection, GFP-positive cells started showing up mainly in the trabecular meshwork region (A). Six days after the injection, more GFP-positive cells were found in the cornea (B). Fourteen days after the injection, GFP signals disappeared from the trabecular meshwork (TM) (C). Twenty three days after injection, almost all GFP signals disappeared (D). Live image from Z-stack scanning showed that GFP-positive cells could be found all over the cornea six days after injection (S2A Fig). High magnification revealed the morphology of the GFP-positive cells. Hexagon/star-shaped cells found in the cornea were endothelial cells. Mesenchymal cells found in the TM region were trabecular meshwork cells (B). AdGFP and AdWnt5a virus were injected into the anterior chamber of mouse eyes. Two weeks and two months after injection, the eyes were collected and subjected to anti-Wnt5a immunostaining. Two weeks after injection, no positive signal could be found in AdGFP-injected eyes (S3A Fig). Strong positive signal could be found in the trabecular meshwork and corneal endothelial cells of AdWnt5a-injected eyes (B). Two months after injection, both AdGFP-injected eyes and AdWnt5a-injected eyes were negative for Wnt5a (C and D).

### Abnormal macroscopic features of AdWnt5a-injected eyes

**Fig 1:**
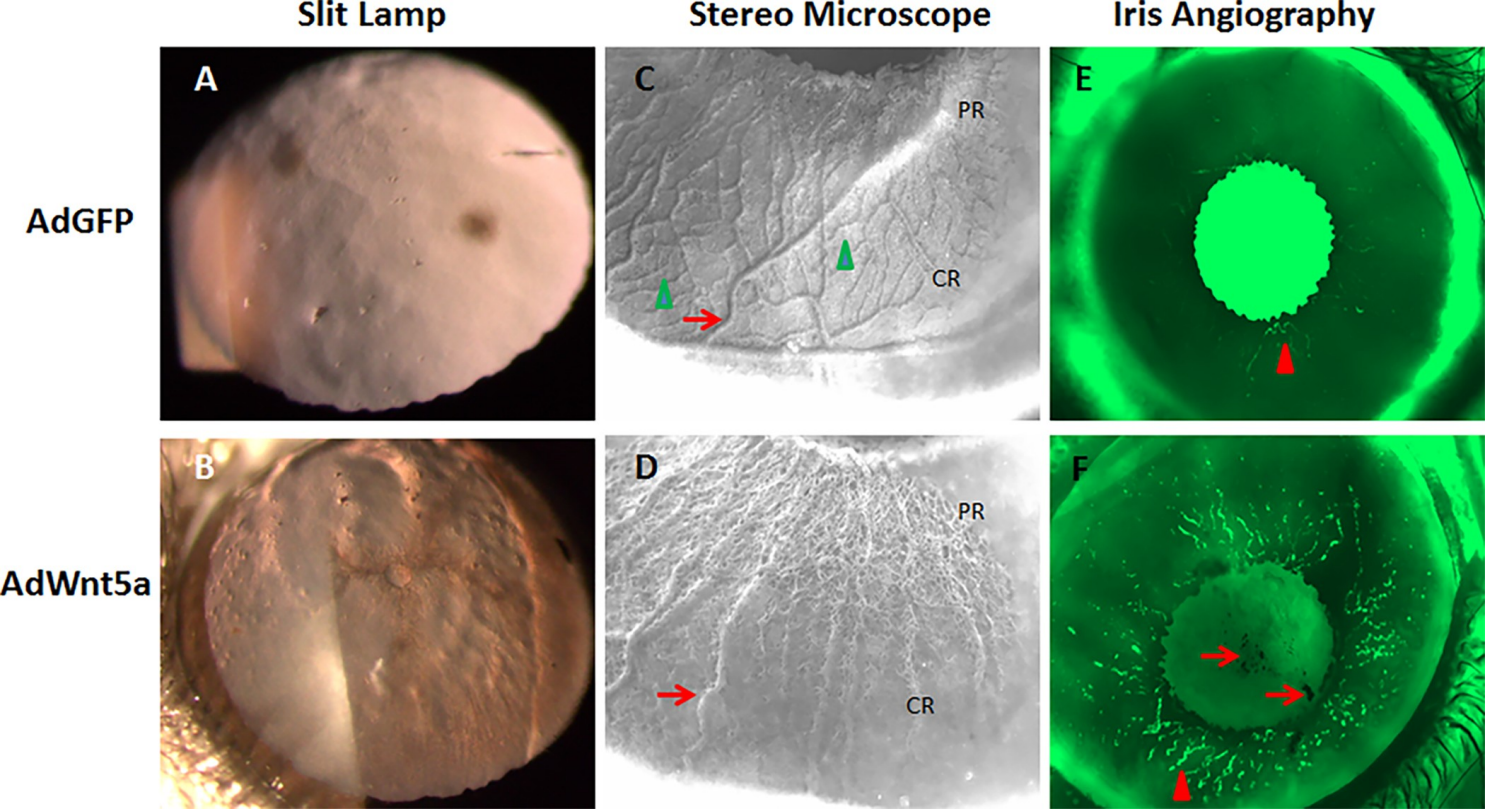
Abnormal macroscopic features of AdWnt5a-injected eyes. C57BL mice were injected with AdGFP or AdWnt5a at four months old. Two months after injection, the mice were examined using a slit lamp and stereomicroscope. Under the slit lamp, the anterior lens capsule of the AdGFP-injected eye appeared normal (A). The anterior lens capsule of AdWnt5a-injected eyes resembled the moon's surface (B). In the center was a crater-shaped structure encircled by dark material. Granular deposits of different sizes were also identifiable. Under the stereomicroscope, the anterior iris surface of the AdGFP-injected eyes was smooth and intact with a complex vascular network clearly visible (C). The vascular network consisted of major radial vessels (Arrow) and secondary branching vessels (Arrow head). The anterior iris surface of AdWnt5a-injected eyes was porous with altered vascular structures. The major radial vessels became bulging and tortuous (Arrow) in the ciliary region and were gradually eroded away by the holes in the pupillary region (D). Iris vasculature was revealed by fluorescein angiography. In AdGFP-injected eyes, most of the blood vessels were blocked by the dark melanin and only a few around the pupillary margin were visible (Arrowhead in E). In AdWnt5a-injected eyes, vascular structures were clearly visible in the pupillary region of the iris in a tortuous pattern (Arrowhead in F). Dark pigments can also be seen in the pupil area (Arrows). Abbreviations: PR = pupillary region, CR = ciliary region.

Under the slit lamp, the anterior lens capsule of AdWnt5a-injected eyes had more topographic surface irregularities than those of AdGPF-injected eyes (compare Fig 1A and 1B), such as granular deposits and radial striations (Fig 1B). Under the stereomicroscope, the anterior iris surface of the control eyes was covered by a smooth and intact anterior limiting layer with well-organized vascular networks underneath. The vascular networks consisted of major radial vessels and a complex of intermediate and small vessel networks (Fig 1C). The anterior surface of the iris from AdWnt5a-injected eyes was rough and had numerous small holes. Bulging, tortuous radial vessels and disintegrated small vascular networks could be observed shallowly buried by the degenerated anterior limiting layer (Fig 1D). Iris angiography verified the disintegration of the iris anterior limiting layer in AdWnt5a-injected eyes. In control eyes, most of the blood vessels were blocked by pigment and only a few vessels could be visualized at the pupillary margin (Fig 1E). In AdWnt5a-injected eyes, vascular structures were clearly visible throughout the pupillary zone and segments of vessels were also visible in the ciliary zone. Dark granules were also present in the pupil area and around the pupillary margin of the iris (Fig 1F).

### Abnormal microscopic features of AdWnt5a-injected eyes

**Fig 2:**
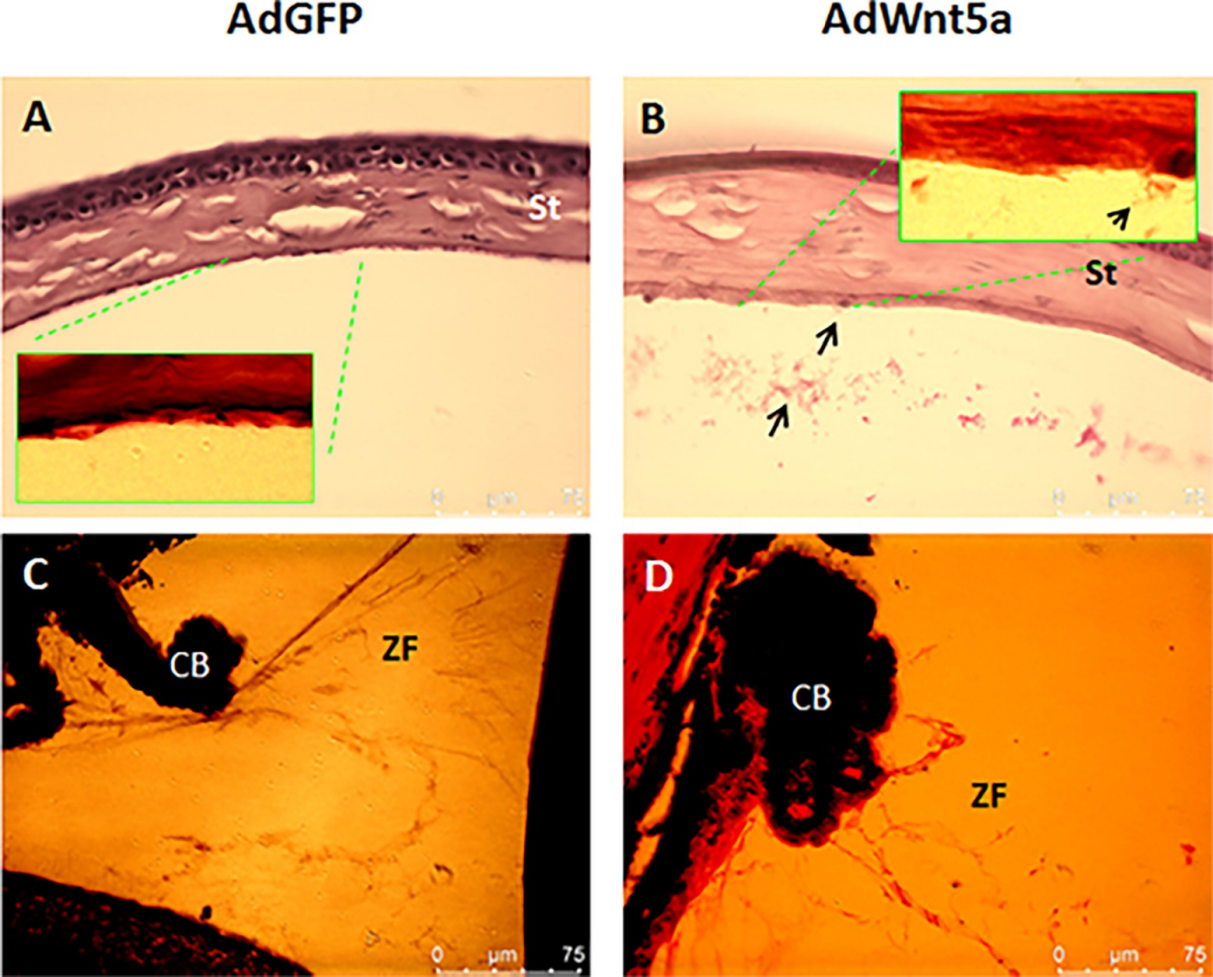
Abnormal microscopic features of AdWnt5a-injected eyes revealed by Hematoxylin and Eosin staining. Two months after adenovirus injection, eyes were collected and processed for pathology analysis. Hematoxylin and Eosin staining revealed the absence of aggregates in the anterior chamber of AdGFP-injected eyes (A). AdWnt5a-injected eyes had fibrillar aggregates in the anterior chamber as indicated by the arrow (B). Zonular fiber structures were also visible after adjustment of color saturation. In AdGFP-injected eyes, the main zonular bundles were thick, straight and well-organized (C). In AdWnt5a-injected eyes (D), the main zonular bundles were disorganized, exhibited lack of tension, and were partially fragmented. Abbreviations: St = stroma. ZF = zonular fiber, CB = ciliary body.

Next, histopathological analyses were conducted to reveal pathological features of AdWnt5a-injected eyes on the light and electron microscopic level. The anterior chamber of control AdGFP-injected eyes was free of extracellular aggregates by H & E staining (Fig 2A), while AdWnt5a-injected eyes contained accumulations of an abnormal flocculent material (Fig 2B). Clumps of this material were found adhering to the apical surface of the corneal endothelial cells and freely floating in the anterior chamber (higher magnification insert of Fig 2B).

The structure of ciliary zonules can be best revealed by overexposing the H & E images. In the control eyes, the main zonular bundles connected the ciliary processes to the anterior lens surface and fan shaped fine bundles attached to the equatorial lens capsule (Fig 2C). In AdWnt5a-injected eyes, the main zonular bundles were disorganized and detached from the lens (Fig 2D). The broken zonular bundles may result from tissue processing but also could be due to an increased weakness and fragility of the zonules.

**Fig 3:**
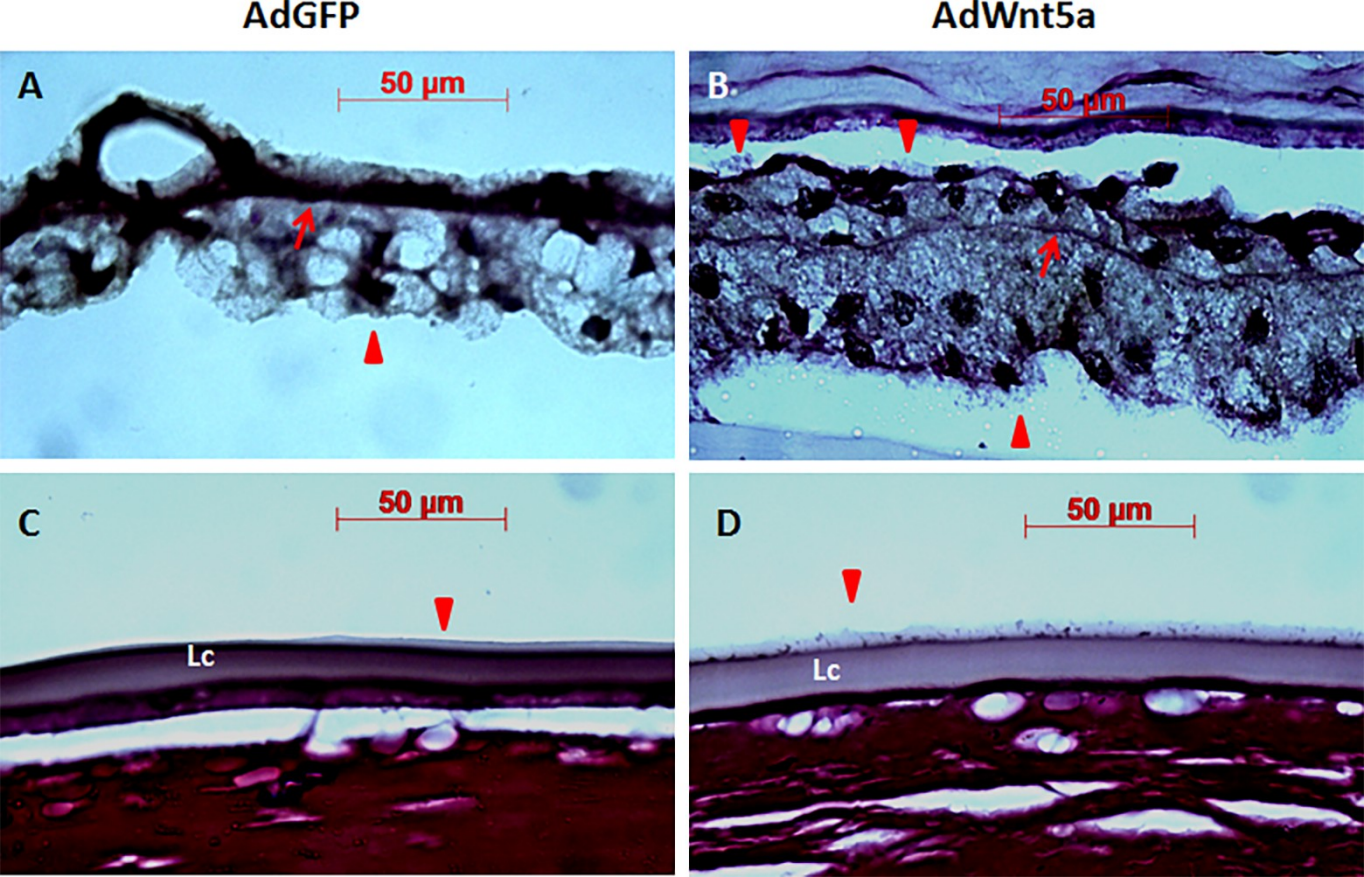
Fibrillary deposits on the iris and the anterior lens capsule. Two months after adenovirus injection, eyes were collected and processed for pathology analysis. After deparaffinization, the slides were treated with 10% hydrogen peroxide in PBS overnight at room temperature to reduce the intensity of dark pigmentation in the iris. Hematoxylin and Eosin staining revealed the tissue structure of the iris. Eosin-positive dilator muscle (arrows in A and B) divided the iris into stroma and epithelium. Anterior to the dilator muscle was iris stroma and posterior was double layers of pigmented epithelial cells. In AdGFP-injected eyes, the iris had well-defined clean edges with no extra material attached to them (A). In AdWnt5a-injected eyes, both anterior and posterior edges of the iris were coated with loose, fibrillary material (arrow heads in B). The surface of anterior lens capsule from AdGFP-injected eye was smooth (C). An extra layer of membrane-like material was visible on top of anterior lens capsule from AdWnt5a-injected eye (arrow head in D). Dark granules can be found randomly embedded in this membrane-like material. Abbreviations: Lc = Lens capsule.

To reveal the tissue structure of the heavily pigmented iris, a de-pigmentation step was used prior to H & E staining. In AdGFP-injected eyes, the iris was separated by the strong Eosin-positive dilator muscle. Anterior to the dilator muscle was iris stroma and posterior to the muscle was a double layer of pigmented epithelial cells. In control eyes, the anterior and posterior surface of the iris were rather smooth and well-defined (Fig 3A). In AdWnt5a-injected eyes, the dilator muscle appeared atrophic, and both anterior and posterior surfaces were covered with fuzzy fibrillary deposits (Fig 3B). In certain places, the posterior iris surface also showed irregular indentations in a saw-tooth like manner (this feature could be seen at the right margin in Fig 3B). The surface of the anterior lens capsule, which appeared rather smooth in control eyes (Fig 3C), was also covered by an abnormal layer of extracellular material containing pigment granules in AdWnt5a-injected eyes (Fig 3D).

**Fig 4:**
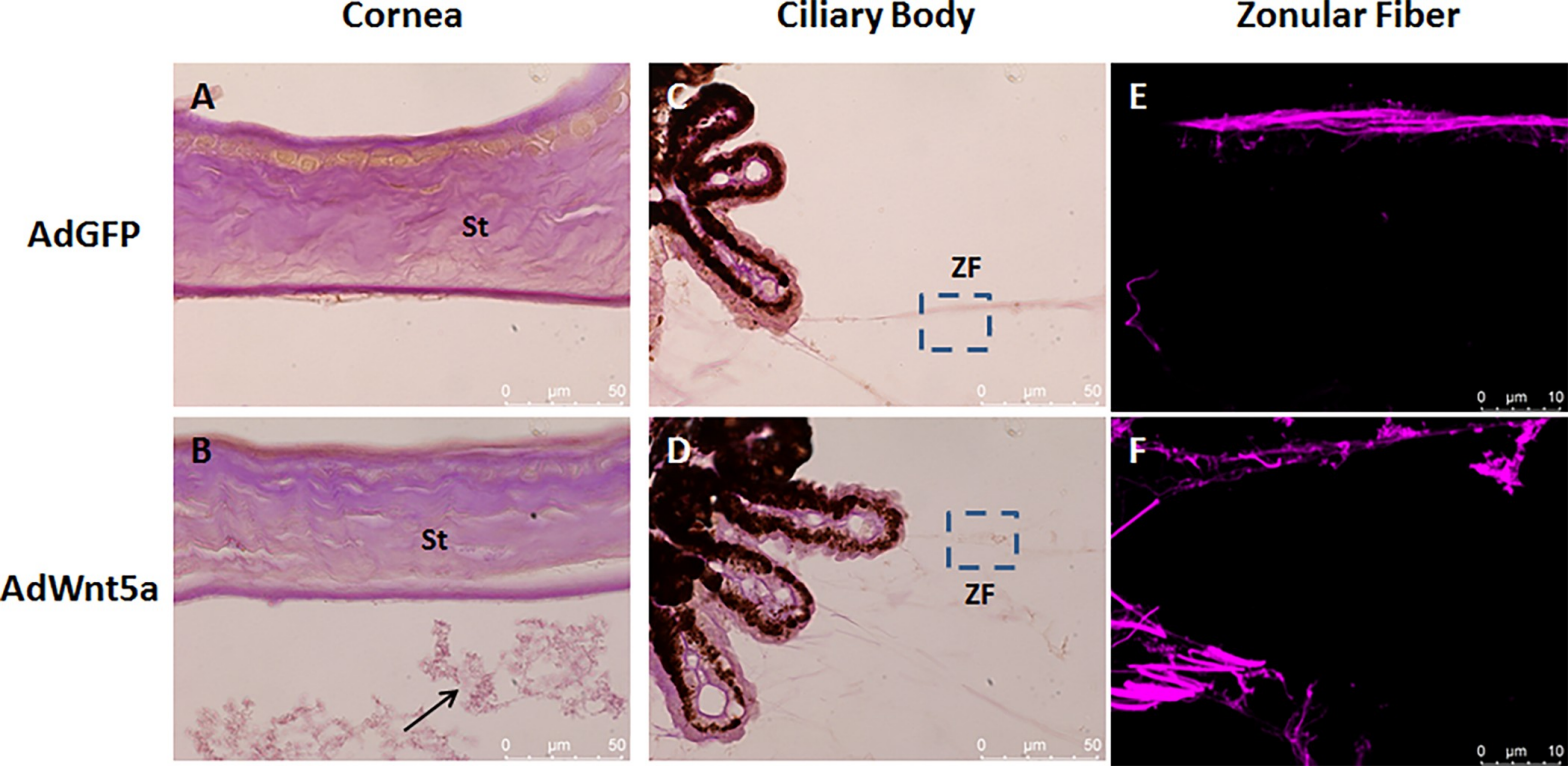
Abnormal distribution of proteoglycan revealed by Periodic acid-Schiff staining in AdWnt5a-injected eye. Periodic acid-Schiff's (PAS) reagent stains the sugar side chains of the proteoglycans. In AdGFP-injected eyes, proteoglycans were abundant in the corneal stroma and the Descemet's membrane (A). In addition to the presence of proteoglycans in the corneal stroma and the Descemet`s membrane, AdWnt5a-injected eyes also contained PAS-positive aggregates in the anterior chamber (Arrow in B). Zonular fibers were also PAS-positive in both eyes (C, D), but confocal fluorescence imaging uncovered different structures of the zonular bundles. In AdGFP-injected eyes, the major zonular bundle consisted of several sub-fibers twisting around each other forming a rope-like structure (E). In AdWnt5a-injected eyes, the sub-fibers of the major zonular bundle were weakly stained and decorated with pink granules (F). Abbreviations: St = stroma, ZF = zonular fiber.

Periodic acid-Schiff's reaction (PAS) staining highlighting structures with a high proportion of carbohydrates, showed positivity of extracellular material aggregates in the anterior chamber of AdWnt5a-injected eyes in contrast to control eyes (Fig 4A and 4B). Zonules of both control and AdWnt5a-injected eyes were also PAS positive (Fig 4C and 4D). To reveal the detailed structure of the zonular fibers, we took advantage of the autofluorescence of the PAS stain [34] and used a confocal fluorescence microscope to scan PAS-positive zonules at a higher magnification (boxed areas of Fig 4C and 4D). As shown in Fig 4E, the main zonular bundles of the control eyes were thick and well-organized. In contrast, the main zonular bundles of AdWnt5a-injected eyes appeared rather rarefied and fragmented (Fig 4F).

**Fig 5:**
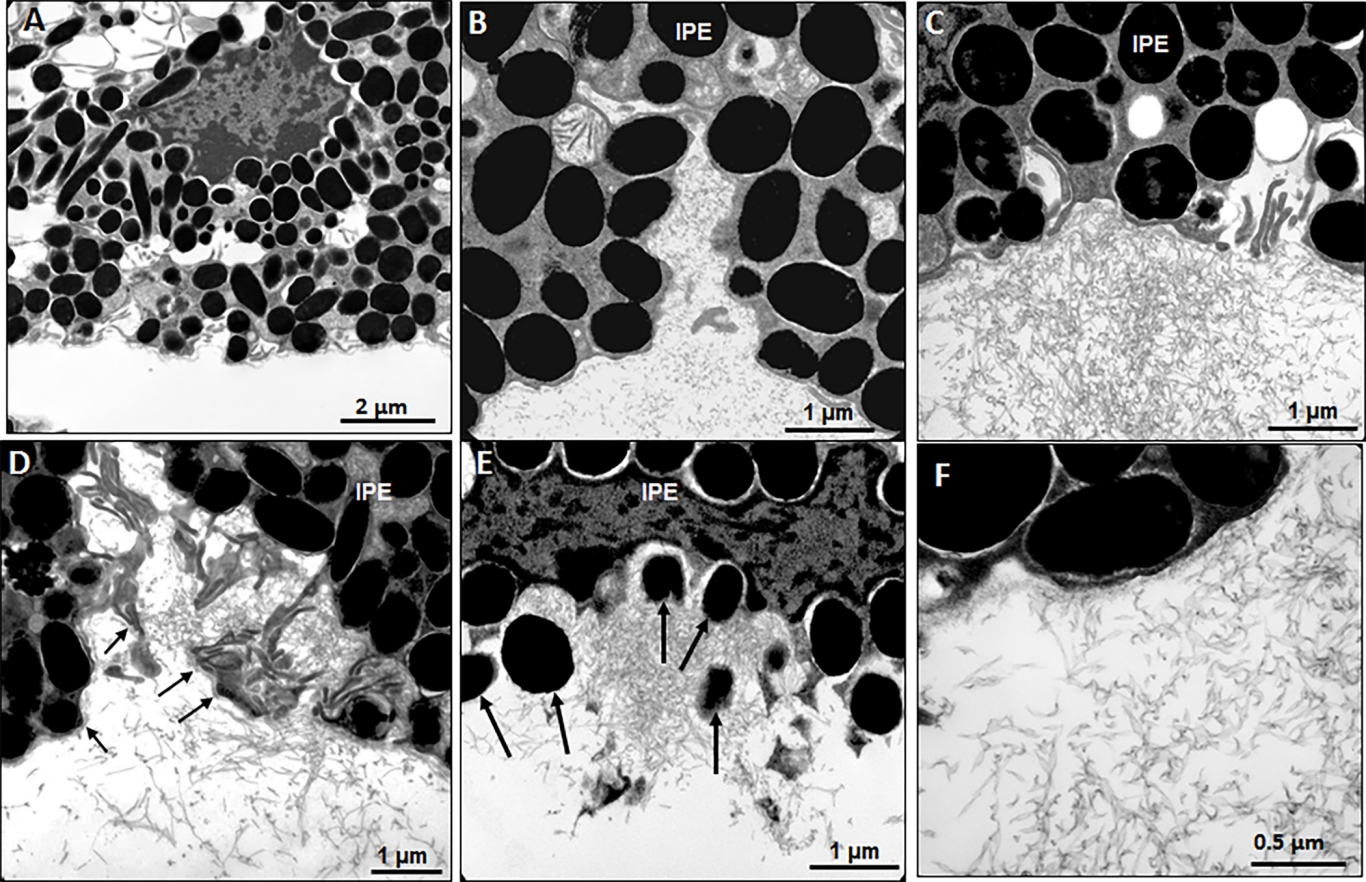
Alterations of iris pigment epithelium by transmission electron microscopy. Electron microscopy of an uninjected eye revealed that the posterior chamber was free of microfibrils (A). Microfibrils emerged from lateral cell invaginations (B), accumulated as aggregates on the epithelial surface (C) and were associated with disruption of the basement membrane (arrows) (D) and liberation of pigment granules (arrows) (E). Ultrastructure analysis revealed that the microfibrils lacked the typical “beads-on-a-string” structure and were different from those found in PEX patients.(F). Abbreviations: IPE = iris pigment epithelium, NPE = nonpigmented ciliary epithelium, St = stroma, Zo = zonular fibrils.

Transmission electron microscopy of uninjected eye showed that a region of posterior chamber adjacent to the iris pigmented epithelium was free of microfibrils (Fig 5A). Lateral surface invaginations between iris pigment epithelial cells appeared to give rise to bundles of microfibrils (Fig 5B). Accumulation of microfibrillar aggregates on the posterior surface of the pigment epithelium (Fig 5C) was associated with interruptions of the basement membrane (Fig 5D) and liberation of pigment granules (Fig 5E). Higher magnification revealed the ultrastructure of the microfibrils, which was different from those found in PEX patients. The microfibrils had fewer granular aggregates and did not form the typical “beads-on-a-string” structure (Fig 5F).

### Immunofluorescence analysis of biochemical alterations

**Fig 6:**
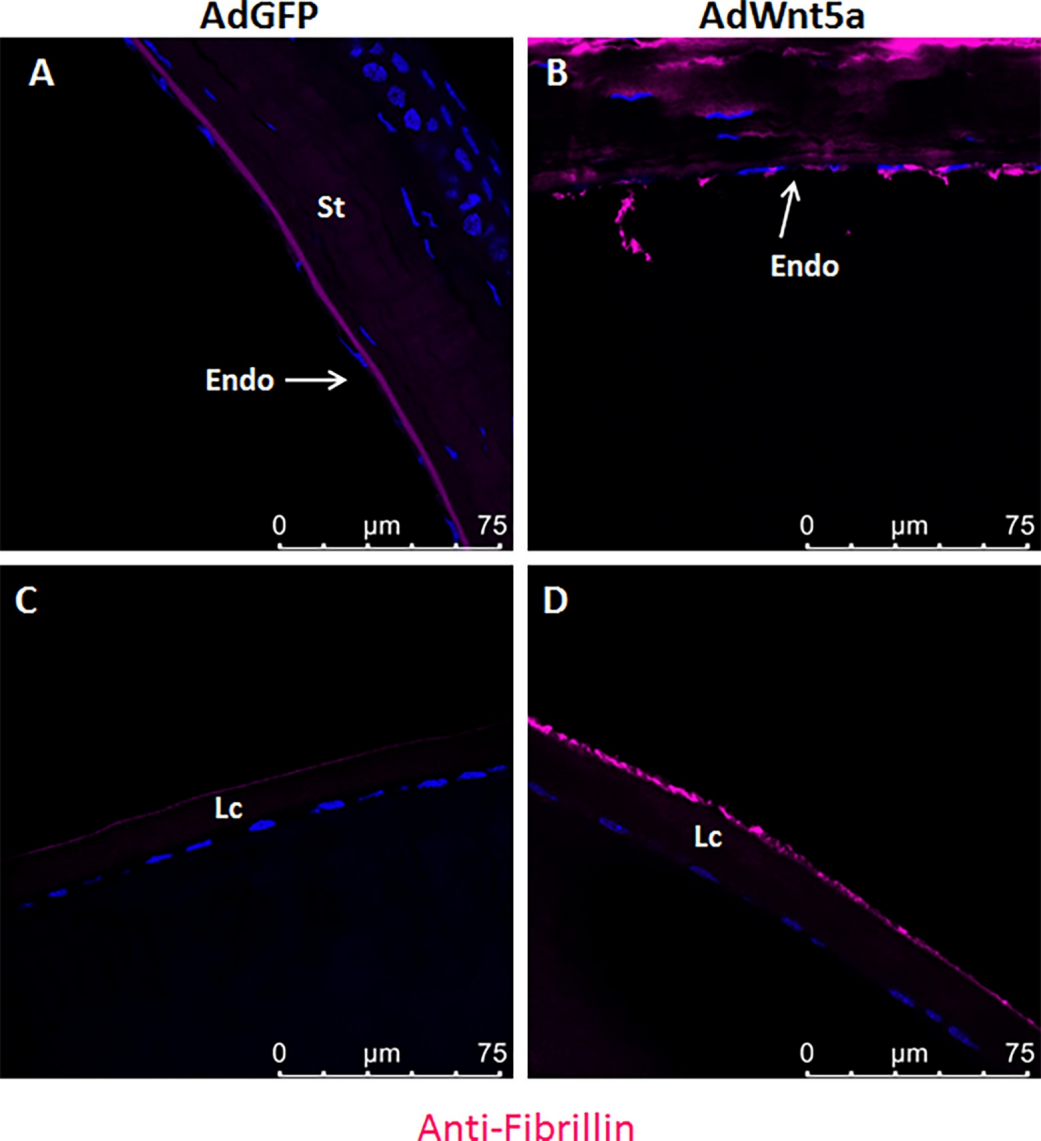
Abnormal distribution of fibrillin-positive material in AdWnt5a-injected eyes. Fibrillin immunostaining was done in eyes two months after adenovirus injection. In AdGFP-injected eyes, positive signals formed a well-organized layer in the Descemet's membrane while the corneal stroma was weakly positive (A). Weak signals were detected on the outer layer of the anterior lens capsule (C). In AdWnt5a-injected eyes, the corneal stroma was strongly positive. The well-organized fibrillin-positive layer was absent from the Descemet's membrane. Fibrillin-positive aggregates were found detaching from the endothelium and floating in the anterior chamber (B). Granular-like positive signals could be found on top of the anterior lens capsule (D). Abbreviations: St = stroma, Lc = Lens capsule, Endo = endothelium.

Next, immunostaining was used to obtain the expression profile of known components of exfoliation material, such as fibrillin and LOXL1. In the control eyes, positive staining for fibrillin was observed in Descemet’s membrane only, whereas corneal endothelium, corneal stroma and lens capsule were largely negative (Fig 6A and 6C). In contrast, AdWnt5a-injected eyes showed markedly increased immunopositivity for fibrillin in the corneal stroma and the corneal endothelium, although Descemet’s membrane was negative (Fig 6B). Fibrillin-positive aggregates were also found attaching to the endothelium and floating in the anterior chamber (Fig 6B). In addition, fibrillin-positive granular deposits could be observed on the surface of the anterior lens capsule (Fig 6D).

**Fig 7:**
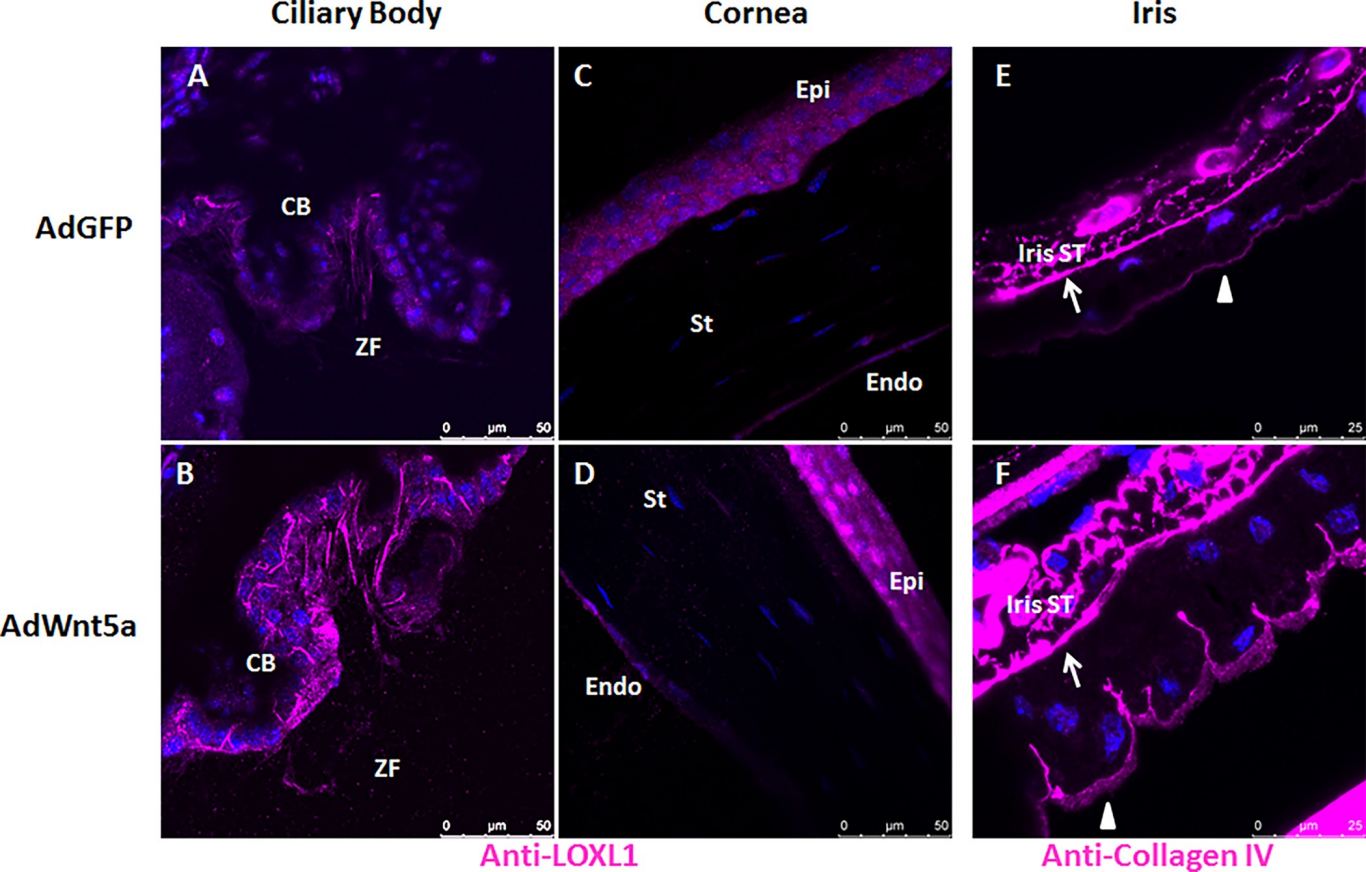
Abnormal distribution of LOXL1 and collagen IV in AdWnt5a-injected eyes. LOXL1 immunostaining was done in eyes two months after adenovirus injection. In AdGFP-injected eyes, LOXL1 was weakly positive in zonular fibers adjacent to non-pigmented ciliary epithelial cells. LOXL1 was also weakly positive in non-pigmented ciliary epithelial cells with an even distribution throughout the cytoplasm (A). In AdWnt5a-injected eyes, there was a strong presence of LOXL1 positive signals in zonular fibers and lateral surfaces between non-pigmented ciliary epithelial cells (B). In control eyes, LOXL1 was evenly distributed within the corneal epithelial cells. The corneal endothelium was also weakly positive (C). In AdWnt5a-injected eyes, strong LOXL1-positive signals were perinuclear or on the posterior pole of the nucleus of the basal corneal epithelial cells. Similar to fibrillin, LOXL1 was found between corneal endothelial cells and on the apical surface of the cells (D). Basement membranes were revealed by anti-collagen IV immunostaining from eyes two months after adenovirus injection. There were two epithelium basement membranes, one between the iris stroma and the anterior epithelium called anterior basement membrane (marked by arrow), another one was for posterior pigment epithelium called posterior basement membrane (marked by arrow head). In AdGFP-injected eyes, basement membrane of the iris posterior epithelium is intact and continuous (A). In AdWnt5a-injected eyes, collagen IV positive signals invaded into the lateral surfaces between the epithelial cells forming the classical saw-tooth structure (B). Abbreviations: CB = Ciliary Body, ZF = Zonular Fiber, Iris ST = Iris Stroma, Epi = Epithelium, St = Stroma, Endo = Endothelium.

In control eyes, weak immunopositivity for LOXL1 could be observed in the non-pigmented ciliary epithelium and zonules (Fig 7A), and in corneal epithelial and endothelial cells (Fig 7C). In AdWnt5-injected eyes, increased LOXL1-positive signals could be observed in the ciliary epithelium and the zonules (Fig 7B), and in nuclei of corneal epithelial and endothelial cells (Fig 7D).

Collagen type IV is the major component of basement membrane. In control eyes, collagen type IV could be immunolocalized to the anterior and posterior basement membranes of the iris pigment epithelium and blood vessels in the iris stroma (Fig 7E). The posterior basement membrane formed a delicate continuous layer on the posterior basal surface of the pigment epithelial cells. In AdWnt5a-injected eyes, collagen IV positive signals were found to extend into lateral surface invaginations between pigment epithelial cells forming the classical saw-tooth structure (Fig 7F),

## Discussion

Mouse models for PEX syndrome are valuable tools for understanding the molecular pathogenesis of the disease. Currently, the only mouse line which has certain ocular features of PEX syndrome is the LYST mouse, which harbors a spontaneous mutation in the Lysosomal-trafficking regulator (LYST) gene [22]. LYST mice have severe iris transillumination defects which can also be found in most PEX syndrome patients. Here, we present a mouse model that shows certain features of PEX syndrome. The model was created by transient expression of Wnt5a in the mouse anterior segment through Adenovirus-mediated gene expression. In this model, no iris transillumination defect was observed, but iris defects presented as iris stromal atrophy, saw-tooth configuration of the iris pigment epithelium, interrupted basement membrane of iris pigmented epithelial cells, accompanied by liberation of pigment granules and production of microfibrillar bundles. Other features observed in this model include the presence of PAS-positive fibrillar material in the anterior chamber; weakened zonular fibers decorated with granular material; and pathological distribution of fibrillar material that is positive for fibrillin and LOXL1, known components of exfoliation material. These features demonstrate that this model recapitulates certain ocular manifestations of human PEX syndrome and indicate a potential involvement of dysregulated Wnt signaling in pathophysiology.

For now, it is unclear how Wnt5a may cause the pseudoexfoliation-like phenotype in our mouse model. From the information about the role of Wnt5a in other diseases, we speculate that the pro-fibrotic effect of Wnt5a may be the culprit. Initially, we found an up-regulation of Wnt5a in the corneal stroma following overexpression of TGF-alpha in the corneal epithelium, which led to secondary angle-closure glaucoma [23]. The same inducible TGF-alpha transgenic mouse line was originally used to study lung fibrosis [35], suggesting that the pro-fibrotic effect of TGF-alpha was associated with Wnt5a up-regulation. As a matter of fact, Wnt5a is one of the major pro-fibrotic factors involved in fibrosis in multiple tissues [36–38]. It has been established that the hallmark of PEX syndrome is extensive fibrotic modulation of extracellular matrix [39].

*LOXL1* is the most important gene in PEX syndrome pathogenesis, though the precise molecular mechanism of how carriers of high-risk *LOXL1* alleles are susceptible to the disease is still unclear. We do not know if LOXL1 plays a role in our mouse model but several interesting observations may be worth noting. In normal mouse eyes, low levels of LOXL1 can be detected in the corneal epithelial cells. In AdWnt5a-injected eyes, LOXL1 protein can be found clustering around the posterior pole of the nucleus of basal corneal epithelial cells. It is important to note that the antibody we used, as is the case with most commercial anti-LOXL1 antibodies, is directed against the propeptide region of the protein because the sequence of the catalytic domain is conserved among all lysyl oxidase isoforms. It is unlikely that the lysyl oxidase activity of LOXL1 towards extracellular matrix proteins plays an important role in PEX syndrome pathogenesis [40] because the PEX-associated non-synonymous risk alleles lead to amino acid changes in the propeptide region. Proteomic analysis of the exfoliation material from patients also revealed that the majority of LOXL1 peptides identified were from the propeptide region [14]. The role of the propeptide is to deliver the protein to the extracellular matrix where it can be processed by BMP-1 or procollagen C proteinase (PCP) into the mature enzyme. Studies on how and where high-risk allele LOXL1 proteins are processed will shed light on the pathogenesis of PEX syndrome.

Although our model recapitulated certain ocular features of human PEX syndrome, several characteristics of PEX syndrome are not found in this model. First, there is no iris transillumination defect in this model, although liberation of pigment granules from the iris pigment epithelium could be seen on electron microscopy. The presence of collagen IV in the basolateral surface of iris pigment epithelial cells suggests the loss of cell junctions. However, the beginning saw-tooth configuration of the iris pigment epithelium may be the preceding event to further loss of pigment and epithelial cells leading to macroscopically evident transillumination defects. Another difference between this model and human PEX is the lack of a typical pattern of exfoliation deposit on the anterior lens capsule, i.e., a translucent central zone of the size of the undilated pupil, a clear intermediate zone and a granular peripheral zone [41]. In our model, we can observe only a significant increase of granular deposition on the anterior lens surface. To our knowledge, the typical pattern of human exfoliation deposits on the anterior lens capsule has not been observed in any of the mouse models so far. This may be due to the lack of accommodation in rodent eyes. The pattern observed in our model may represent a pre-granular stage of PEX syndrome [42]. Thirdly, the microfibrils found in this model were different from those found in PEX patients, which consist of smaller granular aggregates forming “beads-on-a-string” structure. This suggests that besides Wnt5a activation, other factors, such as lysosomal dysfunction and/or high-risk LOXL1 variants are essential for modeling the disease in mice. Finally, the model has drawbacks of Adenovirus-mediated gene expression. The Wnt5a expression only lasts about three weeks after injection, while many PEX-associated features, including glaucoma development, may take much longer to develop. Most of the observations are possibly secondary to Wnt5a expression. Because of a lack of good anti-Wnt5a antibody to detect endogenous Wnt5a expression, we do not know if endogenous Wnt5a expression is up-regulated. A tissue-specific inducible Wnt5a transgenic mouse line would be able to maintain long term Wnt5a expression and eventually may be helpful in determining if Wnt5a can also cause glaucoma and the relationship between glaucoma and PEX syndrome.

In conclusion, we observed certain ocular features of PEX syndrome in mice with transient Wnt5a expression. There are some common characteristics among human PEX syndrome, LYST mice, and AdWnt5a-injected mice. They are all associated with a pronounced pathology of the iris and production/accumulation of fibrillar extracellular matrix. More studies are needed to examine the potential relationship among lysosomal dysfunction, LOXL1 maturation and Wnt5a activity.

## Supporting information

S1 Fig Time course of Adenovirus-mediated gene expression after intracameral injection. Adenovirus (GFP/Wnt5a co-expression) was injected into the anterior chamber. The same eye was imaged by the fluorescence stereomicroscope at different time point. Three days after the injection, GFP-positive started showing up mainly in the trabecular meshwork region, forming a “green ring” around the cornea. The injection site was still visible at 4 o`clock region (A). Six days after the injection, more GFP-positive cells were found in the cornea, and later confirmed to be corneal endothelial cells. The injection site was in the process of healing (B). Fourteen days after the injection, GFP signal disappeared from the trabecular meshwork (C) and the injection site was completely healed. Twenty three days after injection, almost all the GFP signal disappeared.
(TIF)

S2 Fig Trabecular meshwork cells and corneal endothelial cells were the target cell of Adenovirus-mediated gene expression. Six days after the Adenovirus (GFP/Wnt5a co-expression) injection, live image was taken by fluorescent stereomicroscope using Z-stack scanning. GFP-positive cells can be found all over the cornea. Cornea in the pupil region should also be positive but was overwhelmed by the reflect green light (A). High magnification revealed the morphology of the GFP-positive cells. Hexagon/star-shaped cells found in the cornea were endothelial cells. Mesenchymal cells found in the TM region were trabecular meshwork cells (B).
(TIF)

S3 Fig Wnt5a expression in the cornea after adenovirus intracameral injection. AdGFP and AdWnt5a virus were injected into the anterior chamber of mouse eye. Two weeks and two months after injection, the eyes were collected and subjected to anti-Wnt5a immunostaining. Two weeks after injection, no positive signal can be found in AdGFP-injected eye (A). Strong positive signal can be found in the trabecular meshwork and corneal endothelial cells of AdWnt5a-injected eye (B). Two months after injection, AdGFP-injected eye was negative for Wnt5a (C). In AdWnt5a-injected eye, strong positive signal in the endothelial cells disappeared. The overall signal was stronger than that of the control cornea but need to be verified by a secondary detection method such as in situ hybridization.
(TIF)
