# Supplementary figures and images for: Transient expression of Wnt5a elicits ocular features of pseudoexfoliation syndrome in mice
Source: PLoS One. 2019 Mar 6;14(3):e0212569. doi: 10.1371/journal.pone.0212569 (PMC6402630; doi:10.1371/journal.pone.0212569)

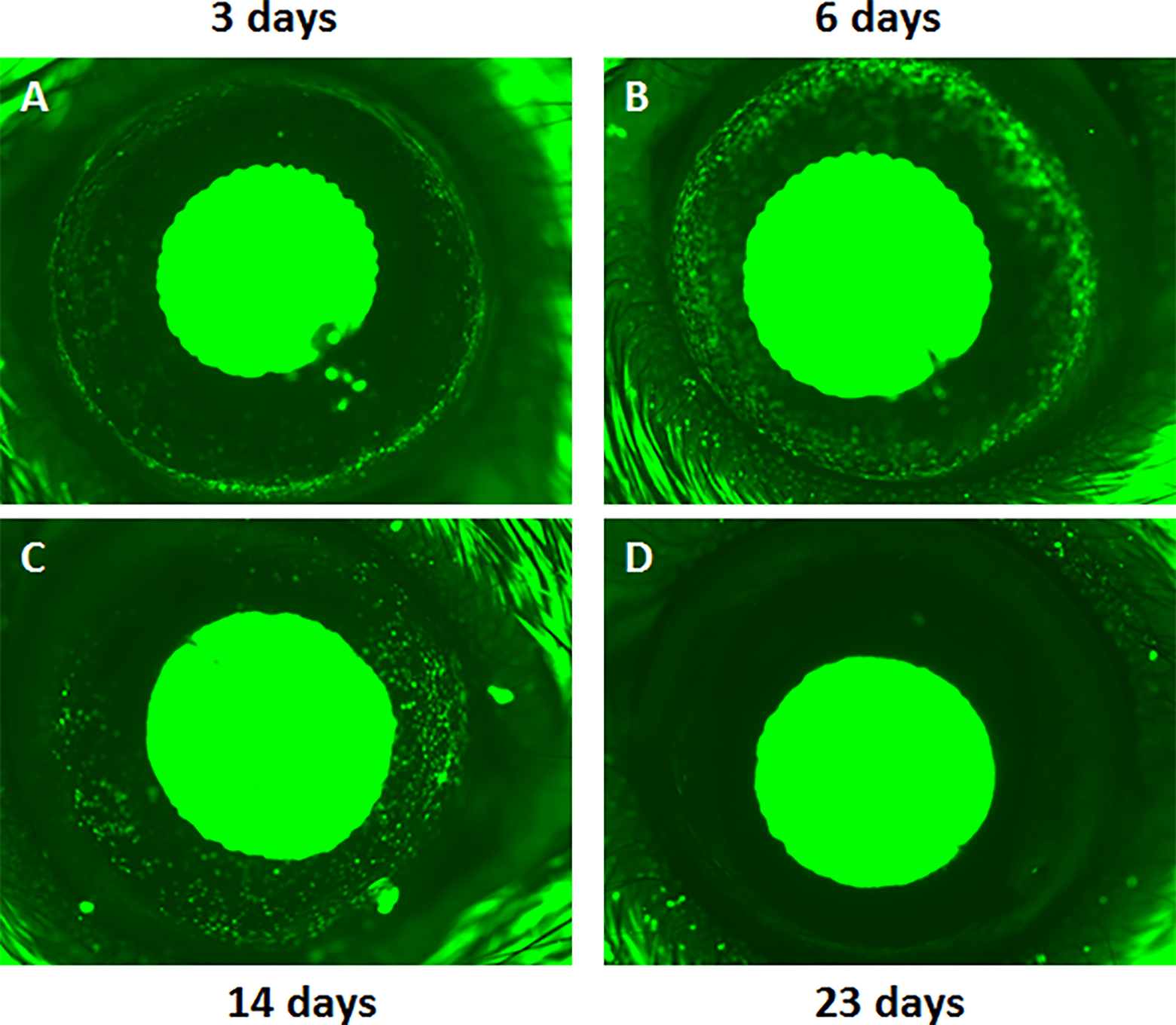

Supplement: S1 Fig — Adenovirus (GFP/Wnt5a co-expression) was injected into the anterior chamber. The same eye was imaged by the fluorescence stereomicroscope at different time point. Three days after the injection, GFP-positive started showing up mainly in the trabecular meshwork region, forming a “green ring” around the cornea. The injection site was still visible at 4 o`clock region (A). Six days after the injection, more GFP-positive cells were found in the cornea, and later confirmed to be corneal endothelial cells. The injection site was in the process of healing (B). Fourteen days after the injection, GFP signal disappeared from the trabecular meshwork (C) and the injection site was completely healed. Twenty three days after injection, almost all the GFP signal disappeared. (TIF) [file pone.0212569.s001.tif]

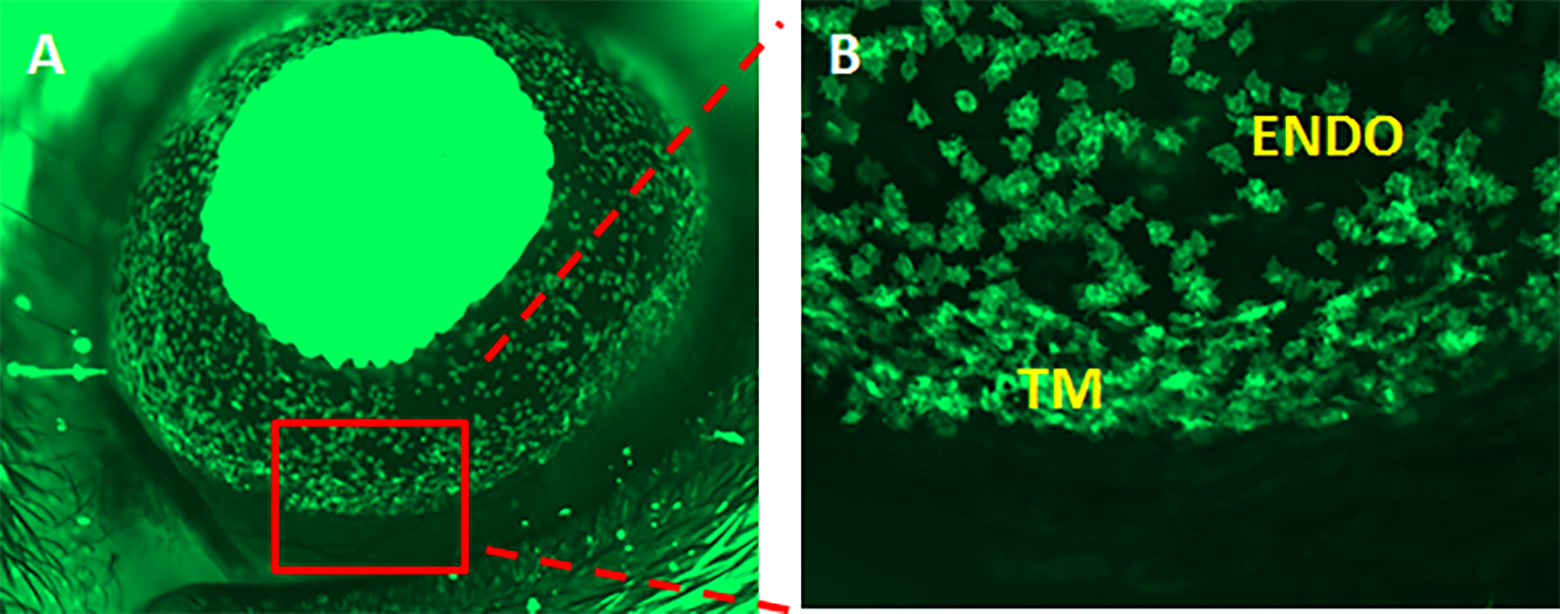

Supplement: S2 Fig — Six days after the Adenovirus (GFP/Wnt5a co-expression) injection, live image was taken by fluorescent stereomicroscope using Z-stack scanning. GFP-positive cells can be found all over the cornea. Cornea in the pupil region should also be positive but was overwhelmed by the reflect green light (A). High magnification revealed the morphology of the GFP-positive cells. Hexagon/star-shaped cells found in the cornea were endothelial cells. Mesenchymal cells found in the TM region were trabecular meshwork cells (B). (TIF) [file pone.0212569.s002.tif]

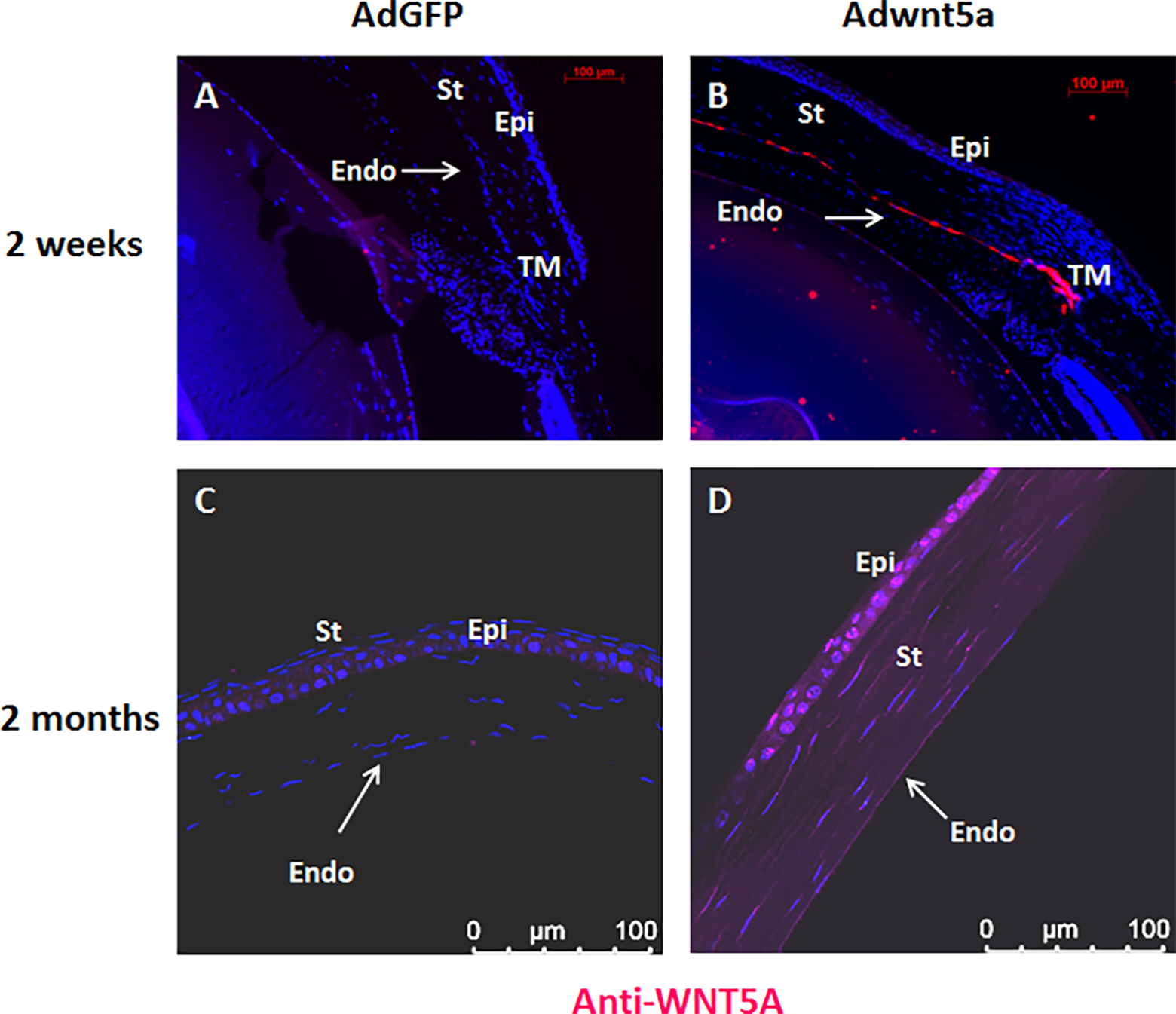

Supplement: S3 Fig — AdGFP and AdWnt5a virus were injected into the anterior chamber of mouse eye. Two weeks and two months after injection, the eyes were collected and subjected to anti-Wnt5a immunostaining. Two weeks after injection, no positive signal can be found in AdGFP-injected eye (A). Strong positive signal can be found in the trabecular meshwork and corneal endothelial cells of AdWnt5a-injected eye (B). Two months after injection, AdGFP-injected eye was negative for Wnt5a (C). In AdWnt5a-injected eye, strong positive signal in the endothelial cells disappeared. The overall signal was stronger than that of the control cornea but need to be verified by a secondary detection method such as in situ hybridization. (TIF) [file pone.0212569.s003.tif]
